# University of Louisville

**Published:** 1849-10

**Authors:** 


### THE WESTERN JOURNAL.

THIRD SERIES.—VOL. IV.—NO. IV.

LOUISVILLE, OCTOBER 1, 1849.

UNIVERSITY OF LOUISVILLE.

Clinical lectures are now delivered six mornings in the week, at the Marine Hospital, to the medical students of the University of Louisville. The Dissecting Rooms are also open to students, and classes are already engaged there. Several courses of lectures preliminary to the winter course are about to be commenced. The regular lectures in the institution will begin on the first Monday in November. The number of students in the city is as large as ever it was thus early in the season.

